# The Beyond the Books Program: Improving Medical Student Attitudes Toward the Underserved

**DOI:** 10.1089/heq.2018.0015

**Published:** 2018-06-01

**Authors:** Aaron M. Briggs, Stephen Y. Wang, Subasish Bhowmik, Jacob Wasag, Roshini C. Pinto-Powell

**Affiliations:** Geisel School of Medicine at Dartmouth, Hanover, New Hampshire.

**Keywords:** attitudes, education, medicine, underserved

## Abstract

**Purpose:** To determine the impact of the Beyond the Books (BTB) program, a short-term pre-clinical intervention, on medical student attitudes toward the underserved (MSATU).

**Methods:** BTB was evaluated through a prospective cohort study using the validated MSATU questionnaire.

**Results:** There were no significant MSATU total score differences between BTB students (*n*=13) and student controls (*n*=29) at the beginning of the program. At the program's conclusion, BTB participant MSATU total scores were significantly higher than those of controls (*p*<0.001).

**Conclusion:** Although limited by selection methods, our MSATU data support the capability of short-term pre-clinical interventions to significantly improve medical student attitudes toward underserved communities.

## Introduction

The United States is currently challenged by striking disparities in health: from infant mortality to cardiovascular disease, traditionally underserved communities in America experience disproportionately high rates of morbidity and mortality across a spectrum of pathology.^1,2^

Despite recommendations from the Association of American Medical Colleges and the Liaison Committee on Medical Education, medical schools in the United States have continued to graduate students lacking the preparation necessary to effectively intervene on behalf of underserved communities.^2–5^ Multiple studies indicate that U.S. resident physicians possess a low level of knowledge regarding issues relevant to medically underserved populations as well as health and healthcare disparities.^6,7^ Numerous studies have also found medical education to negatively influence student attitudes toward the underserved, with commitment to underserved communities observed to be greater when students enter medical school than when they leave.^8–11^

Currently among medical schools in the United States, there exist a limited number of programs designed to prepare students to address health disparity. Of these programs, long-term, multiyear interventions have received the most study.^11,12^ Short-term pre-clinical programs represent a feasible alternative for medical schools that are interested in expanding social medicine curricula but lack the resources necessary for a long-term program. Unfortunately, short-term pre-clinical programs have not been the subject of rigorous evaluation. This study aims to contribute to the literature in this way.^13^

## Methods

### Program description

Beyond the Books (BTB) is an 8-month elective course offered during the first year of undergraduate medical education, which combines classroom didactics with experiential community-based learning. The experiential component of the BTB curriculum seeks to foster a deep sense of empathy and understanding for the challenges experienced by underserved and stigmatized populations by partnering students with underserved members of the Upper Valley community. These individuals, known as BTB community mentors hail from a variety of nonprofit organizations that serve local underserved communities. Students are required to meet with their community mentor once per month and submit reflections regarding what they have learned from their partner. The primary objective of the didactic component of the BTB curriculum is to equip medical students with an understanding of health disparity and the elements that drive it. To this end, BTB utilized a variety of learning formats including guest lectures, panelist discussions, journal article discussions, workshops, and small group discussions (see Supplementary Appendix).

The BTB program was introduced to all Geisel School of Medicine first year students in August of 2016. Thirteen student applicants were selected for participation according to demonstrated desire to learn about health disparity and to work with underserved communities as a physician.

### Program evaluation

The effect of the BTB curriculum on student attitudes toward the underserved was evaluated through a longitudinal prospective cohort study utilizing the medical student attitudes toward the underserved (MSATU) questionnaire.^10^ The MSATU is a validated self-reported measure of medical students' attitudes with regard to providing medical care to underserved populations. We used the first two sections of the instrument as only the first two sections are used to calculate the total score. Section 1 consisted of 23 questions pertaining to the responsibility of physicians, the government, medical students, and charitable organizations to provide healthcare services to indigent populations. Section 2 consisted of 14 questions regarding access to basic and expensive medical care. The MSATU questionnaire was administered to BTB participants (*n*=13) and nonparticipant first year medical student controls (*n*=29) at the initiation and conclusion of the program. First year medical student controls were recruited to take part in the study through class-wide e-mails. Each student who completed the MSATU was compensated 20 dollars for participation. The effect of the BTB curriculum on student understanding of social determinants of health, health disparity, personal bias, and empathy was assessed through a quantitative 5-point Likert scale (strongly disagree to strongly agree) exit survey distributed to BTB participants at the end of the program. Dartmouth IRB board approval (STUDY00029630) was obtained for this study and consent was given by all student participants.

### Statistical analysis

Two-sided independent samples *t*-tests were used to analyze the MSATU mean *t*-scores between cohorts for each of the scales and subscales. Two-sided paired samples *t*-tests were conducted to analyze the MSATU mean *t*-scores within cohorts for each of the scales and subscales. For each scale and subscale, *t*-scores were calculated to have a mean of 50 and a standard deviation of 10. Descriptive statistics were used to evaluate the responses to our exit surveys. We set significance at *p*<0.05. Analyses were conducted using STATA 12.

## Results

The BTB cohort comprised a larger percentage of women relative to the control cohort (85% vs. 62%) and was more ethnically diverse (54% Caucasian and 15% African American vs. 69% Caucasian and 0% African American). In addition, a greater percentage of students in the BTB cohort have been involved in projects providing care to the medically needy (85% vs. 52%) (Table 1).

**Table 1.** **Demographic Information for Beyond the Books Intervention Cohort Versus Medical Student Control Cohort**

Demographic information	Intervention arm (*n*=13)	Control arm (*n*=29)	*p*
Gender, *n* (%)			
Male	2 (15)	11 (38)	0.144
Female	11 (85)	18 (62)	
Average age, years	25.6	24.9	0.43
Race/ethnicity, *n* (%)			
Caucasian	7 (54)	20 (69)	0.065
African American	2 (15)	0	
Other	3 (23)	9 (21)	
Unanswered	1 (8)	0	
Have been involved in projects providing care to the medically needy, *n* (%)	11 (85)	15 (52)	0.060

### Exit survey

Eighty-five percent (11/13) of BTB student program participants responded to the exit survey. These students acknowledged that BTB's curriculum had helped them empathize with underserved communities (mean score 4.4/5) and learn more about social determinants of health and health inequity (mean score 4.3/5). Students also acknowledged that BTB had taught them more about the barriers to health and health challenges that underserved communities face (mean score 4.1/5), helped them identify and correct false stereotypes and preconceptions (mean score 4.2/5), and improved their abilities to interact with patients in a culturally and socially sensitive and thoughtful manner (mean score 4.2/5). Furthermore, students indicated a belief that their participation in BTB will help them to better serve underserved communities as a physician (mean score 4.3/5).

### MSATU survey

**FIG. 1.**
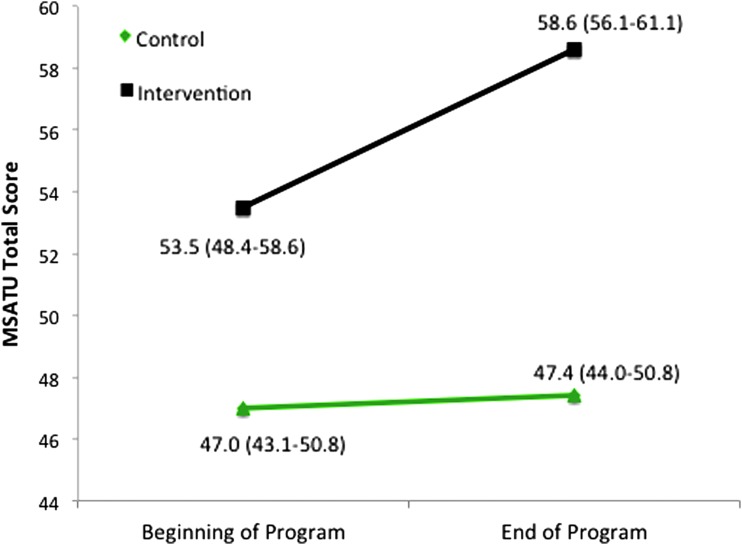
Intervention and Control Cohort Means for MSATU Total Score from Beginning to End of Program: When comparing the intervention group (*black*) with the control group (*green*), independent sample *t*-tests showed no significant difference between the MSATU scores at the beginning of the program (*p*=0.053) and a significant difference between MSATU scores at the end of the program (*p*<0.001). Bracketed values are 95% confidence intervals. MSATU, medical student attitudes toward the underserved.

One hundred percent (13/13) of BTB students took the MSATU survey, whereas thirty-seven percent (29/79) of the remaining first year students took the MSATU survey as the control group. Although there was not a significant difference between MSATU total scores of the BTB and control cohorts at the beginning of the program (53.5, 95% confidence interval [CI]: 48.4–58.6 vs. 47.0, 95% CI: 43.1–50.8, *p*=0.053), BTB participant MSATU total scores were significantly higher than nonparticipant total scores at the end of the program (58.6, 95% CI: 56.1–61.1 vs. 47.4, 95% CI: 44.0–50.8, *p*<0.001) (Fig. 1). When total score was broken down into scales and subscales, BTB students scored significantly higher in the attitudes scale, societal expectations subscale, services scale, and expensive services subscale than their nonparticipant peers at the conclusion of the program; these scales showed no significant between-group differences at the beginning of the program. BTB students were noted to score significantly higher in the professional responsibility and basic services subscales both at the beginning and at the end of the program relative to controls (Table 2).

**Table 2.** **Means of Scales and Subscales for Medical Student Attitudes Toward the Underserved Questionnaire Comparing Between Groups from Beginning to End of Program (Scored as *t*-Scores, Using a Mean of 50 and Standard Deviation of 10)**

Scale	Control	Intervention	*p*
Total			
Beginning of program	47.0	53.5	0.053
End of program	47.4	58.6	<0.01
Attitudes scale			
Beginning of program	47.9	52.0	0.20
End of program	48.3	56.5	<0.01
Societal expectations			
Beginning of program	48.3	49.3	0.76
End of program	49.6	55.4	<0.05
Professional responsibility			
Beginning of program	48.2	55.3	<0.01
End of program	46.0	57.5	<0.01
Services scale			
Beginning of program	46.9	53.3	0.08
End of program	48.2	57.7	<0.01
Basic services			
Beginning of program	46.6	53.8	<0.05
End of program	48.6	57.1	<0.01
Expensive procedures			
Beginning of program	47.3	52.8	0.17
End of program	48.2	57.2	<0.01

When comparisons were made within each cohort, there were no statistically significant differences between MSATU scores from the beginning to end of the program, although there were improvements in scores in the BTB cohort for each of the scales and subscales with total scores, societal expectations scores, and basic services scores, each approaching significance at *p*<0.1. Within the control group, the professional responsibility subscale showed a decrease in magnitude that approached significance (*p*=0.097) (Table 3).

**Table 3.** **Means of Scales and Subscales for Medical Student Attitudes Toward the Underserved Questionnaire Comparing Within Groups from Beginning to End of Program (Scored as *t*-Scores, Using a Mean of 50 and Standard Deviation of 10)**

Scale	Beginning of program	End of program	*p*
Total			
Control	47.0	47.4	0.7
Intervention	53.5	58.6	0.097
Attitudes scale			
Control	47.8	48.3	0.7
Intervention	52.0	56.5	0.1
Societal expectations			
Control	48.3	49.6	0.4
Intervention	49.3	55.4	0.088
Professional responsibility			
Control	48.2	46.0	0.097
Intervention	55.3	57.5	0.2
Services scale			
Control	46.9	48.2	0.5
Intervention	53.3	57.7	0.2
Basic services			
Control	46.6	48.6	0.4
Intervention	53.8	57.1	0.076
Expensive procedures			
Control	47.3	48.2	0.6
Intervention	52.8	57.2	0.3

## Discussion

Short-term outcomes indicate that BTB is achieving its desired goals in improving medical student attitudes toward the underserved and equipping medical students with a better understanding of health disparity and the needs of underserved communities.

Current data suggest that MSATU scores drop significantly during medical school,^8–10^ with the first major decline in scores occurring between the first and second years of medical education.^9^ In our study, student participants in BTB experienced a significant improvement in MSATU total scores relative to nonparticipant student controls. Contrary to expectations, MSATU total scores within the control group remained fairly stable over the course of the study period. The absence of the previously observed decline in MSATU total score among our control cohort may be due to two reasons: (1) both our intervention arm and control arms had a high proportion of women, who are more resistant than men to a decline in attitudes,^10^ and (2) our program offered events to the entire medical school community.

Our study acknowledges several limitations. First, small sample size reduces the power of our investigation. Second, the nonrandom nature of BTB participant selection confounds our ability to isolate the influence of the BTB curriculum on student attitudes. It should be noted that no significant difference was observed in MSATU score between BTB participants and nonparticipant controls at the beginning of the study; however, this null effect may change with a larger sample size. Finally, due to the brief time course of our study, our evaluation is limited to short-term outcomes. To our knowledge, the literature has not evaluated whether MSATU score predicts physician behavior after graduation or career choices. As BTB was designed with the ultimate goal of influencing physician practice, our continuing research will track the residency matches of BTB student participants to determine whether these students are more likely to match into primary care or work in medically underserved areas relative to their peers.

Overall, we are encouraged by the short-term outcomes of our program. Medical schools have an important role in the effort to mitigate health disparity by equipping future physicians with the knowledge and empathy necessary to intervene on behalf of underserved communities. Although long-term programs may be beyond the reach of some medical schools due to the resources they require, our findings support the capability of short-term interventions to positively impact medical student attitudes and understanding during formative years of medical education.

## Supplementary Material

Supplemental data

## Abbreviations Used

BTB: Beyond the Books
CI: confidence interval
MSATU: medical student attitudes toward the underserved

## References

[1] Fiscella K, Williams DR Health disparities based on socioeconomic inequities: implications for urban health care. Acad Med. 2004;79:1139–1147 15563647 10.1097/00001888-200412000-00004

[2] Smedley BD, Stith AY, Nelson AR Institute of Medicine Committee on Understanding and Eliminating Racial and Ethnic Disparities in Health Care. In: Unequal Treatment: Confronting Racial and Ethnic Disparities in Health Care. Edited by Smedley BD, Stith AY, Nelson AR Washington (DC): National Academies Press (US), 2003 Copyright 2002 by the National Academy of Sciences. All rights reserved 25032386

[3] AAMC Medical Schools Objectives Project. Report I. Learning Objectives for Medical Student Education. Guidelines for Medical Schools. Washington, DC, Association of American Medical Colleges, 1998 10.1097/00001888-199901000-00010 9934288

[4] Functions and Structure of a Medical School. Standards for Accreditation of Medical Education Programs Leading to the MD Degree. Washington, DC, Liaison Committee on Medical Education, 2016

[5] Green AR, Chun MB, Cervantes MC, et al. Measuring medical students' preparedness and skills to provide cross-cultural care. Health Equity. 2017;1:15–22 10.1089/heq.2016.0011 PMC6071879 30283831

[6] Marshall JK, Cooper LA, Green AR, et al. Residents' attitude, knowledge, and perceived preparedness toward caring for patients from diverse sociocultural backgrounds. Health Equity. 2017;1:43–49 28905046 10.1089/heq.2016.0010 PMC5586003

[7] Wieland ML, Beckman TJ, Cha SS, et al. Resident physicians' knowledge of underserved patients: a multi-institutional survey. Mayo Clin Proc. 2010;85:728–733 20675511 10.4065/mcp.2009.0703 PMC2912734

[8] Crandall SJ, Davis SW, Broeseker AE, et al. A longitudinal comparison of pharmacy and medical students' attitudes toward the medically underserved. Am J Pharm Educ. 2008;72:148 19325964 10.5688/aj7206148 PMC2661153

[9] Crandall SJ, Volk RJ, Cacy D A longitudinal investigation of medical student attitudes toward the medically indigent. Teach Learn Med. 1997;9:254–260 16262550 10.1207/s15328015tlm0904_2

[10] Crandall SJ, Volk RJ, Loemker V Medical students' attitudes toward providing care for the underserved. Are we training socially responsible physicians? JAMA. 1993;269:2519–2523 8487415

[11] Ko M, Edelstein RA, Heslin KC, et al. Impact of the University of California, Los Angeles/Charles R. Drew University Medical Education Program on medical students' intentions to practice in underserved areas. Acad Med. 2005;80:803–808 16123457 10.1097/00001888-200509000-00004

[12] Girotti JA, Loy GL, Michel JL, et al. The Urban Medicine Program: developing physician-leaders to serve underserved urban communities. Acad Med. 2015;90:1658–1666 26488566 10.1097/ACM.0000000000000970

[13] Buckner AV, Ndjakani YD, Banks B, et al. Using service-learning to teach community health: the Morehouse School of Medicine Community Health Course. Acad Med. 2010;85:1645–1651 20881688 10.1097/ACM.0b013e3181f08348 PMC3976958

