# Supplementary material for: The Beyond the Books Program: Improving Medical Student Attitudes Toward the Underserved
Source: Health Equity. 2018 Jun 1;2(1):98–102. doi: 10.1089/heq.2018.0015 (PMC6071908; doi:10.1089/heq.2018.0015)
Supplement: Supplemental data [file Supp_Appendix.pdf]

SUPPLEMENTARY DATA

Supplementary Appendix

Beyond the Books Curriculum 2016–2017

Date	Event
**Fall 2016**	
August 16	General Information Meeting, Application Opens
August 26	Application Closes
August 27	Program Applicants Selected
August 28	Program Applicants Notified
August 31	Introduction to Beyond the Books
September 1	Orientation at The Family Place
September 16	Dinner with Residents at Hartford Dismas House
September 19	Screening of *Unnatural Causes: Is Inequality Making Us Sick?*
September 21	Orientation at The Upper Valley Haven
October 20	Dr. Stephanie White Small Group: Implicit Bias and Serious Mental Illness
October 24	Screening of *13th* and Discussion of Mass Incarceration
October 27	Bridges out of Poverty Workshop at DHMC
November 2	Focus Group: Reflections on the Fall Term
**Winter 2016**	
November 14	Discussion of Implications of the 2017 Election
December 7	Gregory Norman Small Group: Needs Assessment of the Upper Valley
December 7	Dinner with Residents at Hartford Dismas House
December 8	Screening of Pati Hernandez's Documentary *It's Criminal* and Panel Discussion on Incarceration
January 4	Beyond The Books / Urban Health Scholars Journal Discussion: Socioeconomic Inequality, Racism and Health
January 11	January Small Group Discussion
January 19	Dr. Manish Mishra Small Group: The Biopsychosocial Model of Substance Use Disorder
January 24	Dr. Donald West Small Group: Addiction - Clinician Perspective
January 25	Dinner with Residents at Hartford Dismas House
January 31	Dinner with Residents at Hartford Dismas House
February 2	Screening of *Walking into the Unknown*, Discussion of Indigenous Health Facilitated by Shawn O'Leary and Dinner Hosted by the Native American House
February 15	February Small Group Discussion: History of Systemic Racism in Housing
February 16	Dr. Cullen Shipman: Indigenous Health - Clinician Perspective
February 22	Focus Group: Reflections on the Winter Term
**Spring 2017**	
March 20	March Small Group Discussion: Music and Social Justice
March 21	Community Partner Led Small Group: Addiction - Patient Perspective
April 12	Dinner with Residents at Hartford Dismas House
April 19	April Small Group Discussion: The Supplemental Nutrition Assistance Program
April 19	Dr. John Turco and Dr. Benjamin Boh Panel Discussion: Transgender Health
April 24	Going Beyond the Books: Tying it All Together
